# A Comprehensive Case Report on Kienbock’s Disease: Diagnostic Hurdles and Surgical Outcomes With Proximal Row Carpectomy

**DOI:** 10.7759/cureus.66829

**Published:** 2024-08-14

**Authors:** Arun Elamurugan, Shanmugasundaram Nirenjen, Arjun Gokulan Manivannan, Narayanan Jaishankar, Chitra Vellapandian

**Affiliations:** 1 Pharmacy/Pharmacology, Sri Ramaswamy Memorial College of Pharmacy, Sri Ramaswamy Memorial Institute of Science and Technology, Chennai, IND

**Keywords:** pharmacotherapy, proximal row carpectomy, lunate bone, avascular necrosis, kienbock's disease

## Abstract

Kienbock's disease is a rare form of avascular necrosis affecting the lunate bone in the wrist, leading to progressive bone necrosis and functional impairment. The disease's rarity and nonspecific early symptoms present significant diagnostic and treatment challenges. We report the case of a 41-year-old female presenting with chronic left wrist pain and restricted movement without a history of trauma. Her symptoms had progressively worsened over 20 days. Laboratory findings showed an elevated erythrocyte sedimentation rate (ESR) of 14 mm/hour and a slightly prolonged clotting time of 3'30'', while other parameters, including C-reactive protein (CRP), electrolytes, renal function, and blood sugar levels, were within normal ranges. Radiographic imaging indicated lunate collapse, joint space narrowing, subchondral cysts, and secondary osteoarthritis, confirming the diagnosis of Kienbock's disease. Given the severity of her condition, a proximal row carpectomy (PRC) was performed, resulting in significant pain relief and improved wrist function. This case underscores the complexities in diagnosing Kienbock's disease, particularly in regions where it is rare, such as India. The Lichtman classification system was essential in guiding treatment decisions, ranging from conservative measures to surgical interventions. This report highlights the importance of accurate diagnosis and individualized treatment plans in managing Kienbock's disease, demonstrating the efficacy of PRC in advanced stages. It contributes valuable insights into the diagnostic and therapeutic strategies for this rare but impactful condition, emphasizing the need for further research into innovative therapies and preventive measures.

## Introduction

In 1910, Robert Kienbock, an Austrian radiologist, presented the first report of aseptic osteonecrosis of the lunate, also known as avascular necrosis of the lunate bone. This condition, also referred to as aseptic or ischemic necrosis of the lunate bone, lunatomalacia, or simply osteonecrosis, is primarily characterized by a lack of blood supply to the lunate bone. Poor bone vascularization is the most frequently reported cause, leading to bone infarction and subsequent mechanical failure [1]. Despite over a century of research, the precise factors contributing to Kienbock’s disease remain unidentified, making it a complex condition to diagnose and treat. Kienbock's disease is a rare condition with a prevalence of only 0.0066%. It predominantly affects men, particularly those between the ages of 20 and 40 [2]. Globally, the condition has been documented across various populations, though the reported incidence and prevalence may vary due to differences in diagnostic criteria and healthcare access. In the United States, the condition is recognized but remains uncommon, with sporadic cases reported in medical literature. National data from India also indicate low prevalence rates, though increasing awareness and advanced imaging techniques may contribute to a rise in reported cases. The rarity of Kienbock's disease contributes to the challenges in studying and understanding its full impact on different populations [3].

Treating Kienbock’s disease is particularly challenging due to several factors. Firstly, the exact pathophysiology of the disease remains unclear, complicating the development of targeted therapies. The condition is marked by lunate osteonecrosis and is often exacerbated by fractures, carpal confusion, and eventually arthritic degeneration. These complications add layers of complexity to both diagnosis and treatment. Additionally, the initial stages of Kienbock’s disease may present with non-specific symptoms, such as wrist pain and swelling, often mistaken for a sprain or other minor injury. This can lead to delays in diagnosis and appropriate intervention. As the disease progresses, patients may experience decreased range of motion, grip strength, and persistent pain. Advanced stages can lead to significant bone collapse and joint degeneration, resulting in chronic pain and functional impairment. The variability in symptom presentation and disease progression necessitates a highly individualized approach to treatment, further complicating management strategies. Furthermore, there is no universally accepted gold standard treatment for Kienbock’s disease, with available options ranging from conservative methods to surgical interventions. For patients with mild symptoms, particularly adolescents, immobilization is a viable treatment option and should be the first choice until the patient reaches 20 years old. Non-surgical treatment options include the use of anti-inflammatory medications, wrist splints, and activity modification to alleviate symptoms and prevent further progression. Conservative management aims to reduce pain and maintain function, though it may not address the underlying vascular insufficiency. Surgical options are considered for patients with more advanced disease or those who do not respond to conservative treatments. Surgical interventions aim to revascularize the lunate, realign the carpal bones, or, in severe cases, perform partial or total wrist fusion to alleviate pain and restore function. The treatment plan is determined based on radiological and clinical signs, with the Lichtman scale being the standardized and widely used radiological classification system for characterizing Kienbock’s disease. This system categorizes the condition into stages based on radiographic findings of lunate collapse and carpal alignment. Additionally, Bain et al. [4] developed a classification system for arthroscopic interventions, which is based on the number of non-functional articular surfaces on the lunate and the surrounding articulations. Surgical techniques may include procedures such as core decompression, vascularized bone grafting, radial shortening osteotomy, and capitate shortening. Each of these procedures has its own indications, advantages, and potential complications, requiring careful patient selection and preoperative planning.

This case report aims to provide a comprehensive overview of Kienbock’s disease, detailing the clinical presentation, diagnostic challenges, and treatment options available. By examining a specific case, we will highlight the diagnostic process, including the use of imaging techniques and classification systems, and discuss the rationale behind the chosen treatment strategy. This report will also explore the outcomes of the treatment, emphasizing both the successes and any complications encountered. Furthermore, this case report will contribute to the existing body of knowledge by discussing potential new therapies and advancements in the understanding of Kienbock’s disease. Through a detailed analysis of this case, we hope to shed light on the complexities of managing this rare condition and provide valuable insights for clinicians and researchers involved in the care of patients with Kienbock’s disease.

## Case presentation

Case scenario

A 41-year-old female patient presented to the orthopedics outpatient clinic of a tertiary care hospital with complaints of left wrist pain and restricted movement persisting for 20 days. She was admitted on July 1, 2024. The patient reported being in her usual state of health until 20 days prior when she began experiencing pain in her left wrist. The pain was insidious in onset and progressively worsened over time. Notably, there was no history of fall, trauma, or fever. Additionally, the patient had no history of hypertension, diabetes mellitus, or any systemic illness. On general examination, the patient was conscious, oriented, and afebrile. On clinical examination, the body temperature was 98.6°F, the pulse rate was 80 beats per minute, blood pressure was 110/70 mmHg, the respiratory rate was 18 breaths per minute, oxygen saturation (SpO2) was observed as 98%, and they were monitored continuously. On systemic examination, the central nervous system and cardiovascular system were normal; there were no added sounds in the respiratory system. On left wrist examination, there was no swelling or tenderness present; a range of motion (ROM) was restricted and painful - supination pronation, flexion-extension radial and ulnar deviation, sensation intact, dorsalis pedals positive.

Urine analysis was performed on day 1, and it showed two to three pus cells, slightly turbid, and bacteria, as shown in Table 1.

**Table 1:** Urine analysis

Urine analysis	Result
Color	Pale yellow
Appearance	Slightly turbid
Specific gravity	1.030
Reaction	6.0
Albumin	Nil
Sugar (random)	Nil
Ketone bodies	Negative
Bile pigments	Negative
Urobilinogen	Normal
Pus cells	2-3 cells
Crystals	Not found
Epithelial cells	1-2 cells
Bacteria	Seen
Crystals	Not found

The hematological examination was done on day 1; every parameter was within the normal range. The erythrocyte sedimentation rate (ESR) was measured on day 2, and it was found to be abnormal (14 mm/hour). Bleeding time was normal with a value of 2’00’’ (2 minutes and 7 seconds), but clotting time was abnormal at 3’30’’ (4 minutes and 11 seconds) on day 2. The C-reactive protein test showed a negative result on day 2, presented in Table 2.

**Table 2:** Hematological investigations

Hematology	Result	Normal range
Hemoglobin	12.9	12-15 g/dL
Total RBC count	4.3	3.8-4.8 million/cumm
Packed cell volume	38	36-46%
Mean corpuscular volume	88	83-101 fL
Mean corpuscular hemoglobin	30	27-32 pg
Mean corpuscular hemoglobin concentration	34	31.5-34.5 g/dL
Red cell distribution width	11.9	11.6-14%
Total WBC count	5,790	4,000-11,000/cumm
Neutrophils	59.4	40-80%
Lymphocytes	31.6	20-40%
Eosinophils	1.7	1-4%
Basophils	0.7	0-2%
Monocytes	0.6	2-10%
Platelet count	275,000	150,000-410,000/cumm
Absolute neutrophil count (ANC)	3,440	2,000-7,000/cumm
Absolute lymphocyte count (ALC)	1,830	1,000-3,000/cumm
Absolute eosinophil count (AEC)	100	20-500/cumm
Absolute basophil count (ABC)	40	20-100/cumm
Absolute monocyte count (AMC)	380	200-1,000/cumm
Erythrocyte sedimentation rate (ESR)	14	0-12 mm/hour
Bleeding time	2’00’’	2 minutes and 7 seconds
Clotting time	3’30’’	4 minutes and 11 seconds

The serology test (anti-HIV, anti-hepatitis C virus (HCV), hepatitis B surface antigen (HBsAg)) was also done, and the results were negative. Electrolytes were monitored on day 2, and it was found to be normal, as mentioned in Table 3.

**Table 3:** Serum electrolytes

Electrolytes	Result	Normal range
Sodium	138	136-145 mmol/L
Potassium	4.2	3.5-5.1 mmol/L
Chloride	106	98-107 mmol/L
Bicarbonate	24	21-31 mmol/L

The renal function test was done on day 2, and it was found to be normal, as discussed in Table 4.

**Table 4:** Renal function tests

Renal function test	Result	Normal range
Serum urea	26	17-43 mg/dL
Blood urea nitrogen (BUN)	12	6-20 mg/dL
Serum creatinine	0.7	0.6-1.2 mg/dL

Random blood sugar (RBS) was normal at 111 mg/dL (range values: 70-140 mg/dL) on day 2. The coagulation test (prothrombin time, activated partial thromboplastin time) was done on day 2, as shown in Table 5.

**Table 5:** Coagulation test

Clotting test	Result
Prothrombin time (control)	14.0 seconds
Prothrombin time (test)	14.4 seconds
Prothrombin time (international normalized ratio)	1.04
Activated partial thromboplastin time (control)	32.0 seconds
Activated partial thromboplastin time (test)	33.2 seconds

A digital chest X-ray was taken on day 1 and showed no significant abnormality. On day 2, the electrocardiogram (ECG) showed normal sinus rhythm. The echocardiogram (ECHO) showed no regional wall motion abnormalities (RWMA) and normal left ventricle systolic function with an ejection fraction (EF) of 64%. A histopathology test was taken on day 6, as mentioned in Table 6.

**Table 6:** Histopathology analysis P/E - pieces examined

Parent exam	Exam name	Results
Small biopsy	Specimen type	Degenerative tissue from the wrist joint
Small biopsy	Macroscopic description	Bottle marked: Tissue with bone from wrist, received multiple grey-white bony fragments (4) with size varying from 0.5 cm in greatest dimension to 1.6 × 1.3 × 0.5 cm. P/E - 3 A: largest bony fragment; B: larger bony fragment; C: smaller and smallest bony fragment.
Small biopsy	Microscopic description	A-C: Sections studied show fragments of bony tissue showing mature bony trabeculate showing dead osteocytes and empty lacunae with intervening areas showing necrosed marrow elements, fibrocollagenous stroma, and adipocytes. No evidence of atypia in sections studied.
Small biopsy	Impression	Features suggestive of necrotic bone tissue - lunate bone

**Figure 1:**
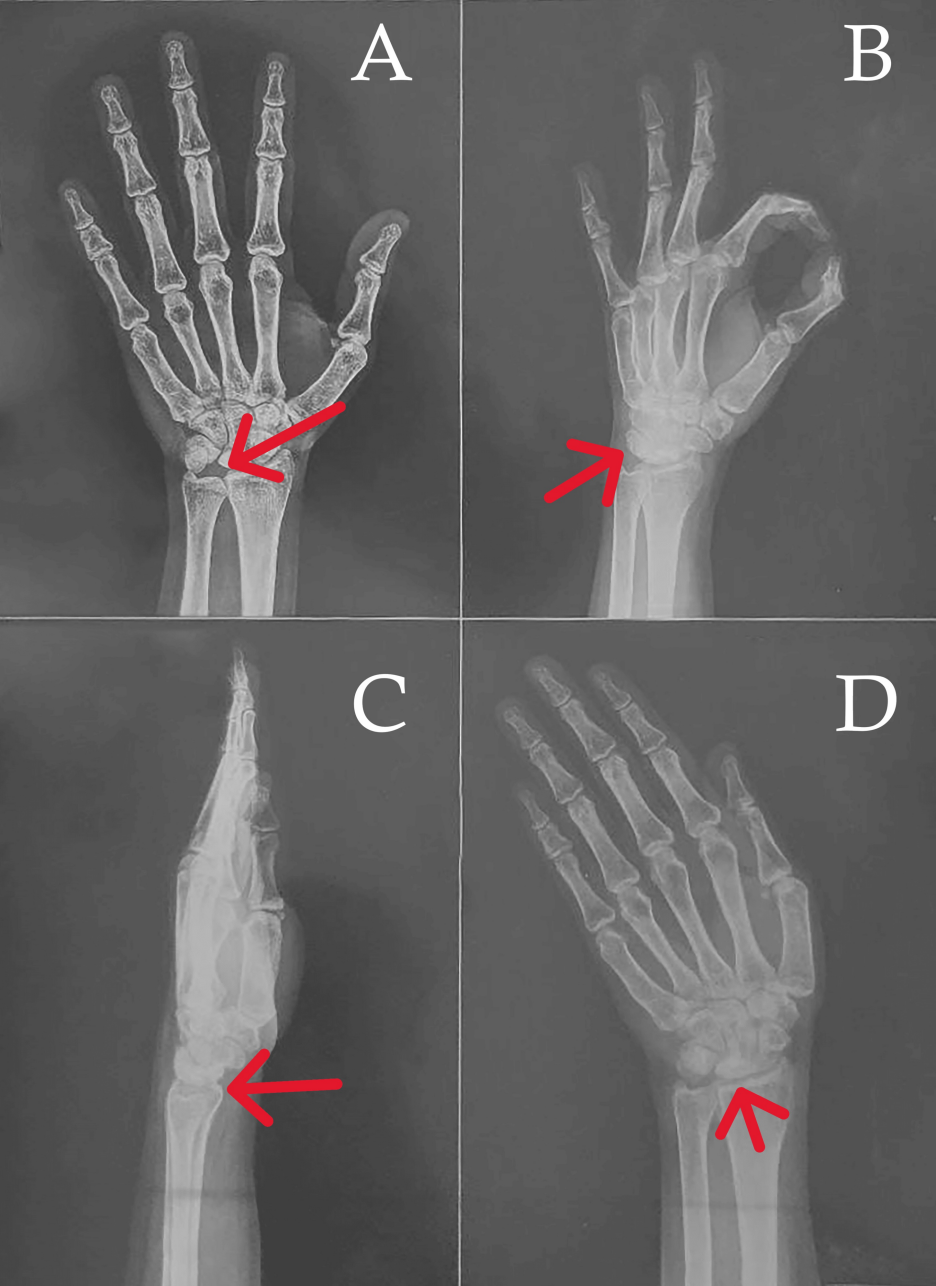
Pre-operative radiological findings A. In the AP view, the lunate bone appears flattened and sclerotic, indicating collapse, with noticeable joint space narrowing and possible secondary osteoarthritis. B. The oblique view further demonstrates the lunate's collapse and fragmentation, consistent with Kienbock's disease. C. The lateral view shows a diminished height of the lunate, reduced joint space, and potential dorsal intercalated segment instability (DISI). D. In the scaphoid view, the lunate's irregular contour and sclerotic appearance further support the diagnosis.

Clinical examination revealed tenderness and significant restriction in wrist movements, although there was no visible swelling, and the neurological and vascular assessments were normal. Initial laboratory investigations, including complete blood count, inflammatory markers, renal function tests, and coagulation profile, were largely within normal ranges, except for a mildly elevated ESR, indicating some level of chronic inflammation. Chest X-ray and ECHO showed no abnormalities. The critical next step in diagnosis is further imaging to assess the extent of the condition. X-ray findings are significant, including evidence of lunate collapse, joint space narrowing, subchondral cysts, and secondary osteoarthritis, confirming the diagnosis of Kienbock's disease. This diagnosis was supported by the clinical picture of persistent wrist pain and restricted motion, combined with the imaging findings, which together provided a comprehensive assessment of the condition, as shown in Figure 1.

Treatment plan

The treatment plan for this patient involved surgery, specifically a proximal row carpectomy (PRC). This procedure is crucial for several reasons: it removes the lunate along with other proximal row carpal bones, thereby alleviating pain, improving wrist function, and preventing further complications associated with Kienbock's disease. Surgical intervention is often required in advanced stages of the disease where conservative treatments are ineffective. Before proceeding with the surgery, cardiology and anesthesia fitness assessments were obtained to ensure the patient could safely undergo the procedure. These evaluations are essential to identify any potential risks and optimize the patient's condition for surgery. On July 2, 2024, the patient underwent a PRC of the left wrist under regional anesthesia. The choice of regional anesthesia provided effective pain control and minimized systemic complications. The surgery aimed to improve the patient's quality of life by reducing pain and enhancing wrist mobility, thereby restoring function and enabling the patient to resume daily activities with improved comfort and capability.

PRC

The patient was positioned supine with the arm placed on an arm board under regional anesthesia. Following aseptic preparation and draping, an 8 cm midline dorsal incision was made. The subcutaneous tissue was carefully dissected to expose and retract the extensor pollicis longus tendon. The dissection proceeded through the plane, splitting the extensor retinaculum until the lunate was identified. The joint capsule was incised, and all ligamentous attachments of the proximal row were severed. Upon inspection, the lunate was found to be fragmented and was subsequently removed. The scaphoid was then identified, its ligaments cut, and the bone excised. Similarly, the triquetrum was identified, its ligaments cut, and the bone removed. Minimal inflammatory tissue was observed, and a thorough wound wash was performed to ensure a clean surgical site. The posterior capsule was re-sutured to the anterior capsule to restore integrity. The subcutaneous tissue was then sutured, and the wound was closed in layers. Finally, a sterile dressing was applied to the surgical site. This procedure will alleviate the patient's chronic wrist pain and improve wrist function, enabling the patient to resume daily activities with increased comfort and mobility [5,6].

Anesthesia record

The patient had no comorbidities or history of trauma, and her cardiac status was stable. A chest X-ray showed no abnormalities, and the serology report was non-reactive. Under aseptic conditions, a supraclavicular block was administered using 30 mL of 0.75% Injection Ropivacaine mixed with 10 mL of distilled water and 30 mcg of Injection Dexmedetomidine. Additionally, 2 g of Injection Cefazolin was given intravenously as a prophylactic antibiotic. The supraclavicular block was chosen to provide effective regional anesthesia, ensuring adequate pain control during and after the surgery. The use of Ropivacaine, a long-acting anesthetic, along with Dexmedetomidine, an adjuvant that prolongs the anesthetic effect and provides sedation, was intended to optimize patient comfort and minimize postoperative pain. The prophylactic administration of Cefazolin was necessary to prevent potential infections, ensuring a safe surgical outcome.

Pre-operative care

The patient was kept nil per oral (NPO) to ensure an empty stomach during surgery, reducing the risk of aspiration. Intravenous fluids were administered with one unit of normal saline (NS) or Ringer's lactate (RL) at a rate of 75 mL/hour to maintain hydration and electrolyte balance. An intravenous dose of 2 g of Injection Cefazolin was administered as a prophylactic antibiotic to prevent surgical site infections, and it was given twice daily. A test dose of 0.2 mL of Injection Lignocaine was given to check for any allergic reactions. Additionally, 0.5 mL of Injection Tetanus toxoid was administered intramuscularly as a precaution against tetanus. Two units of packed red blood cells (PRBC) were reserved to be on standby in case of significant blood loss during the surgery, ensuring preparedness for any intraoperative emergencies.

Post-operative care

**Figure 2:**
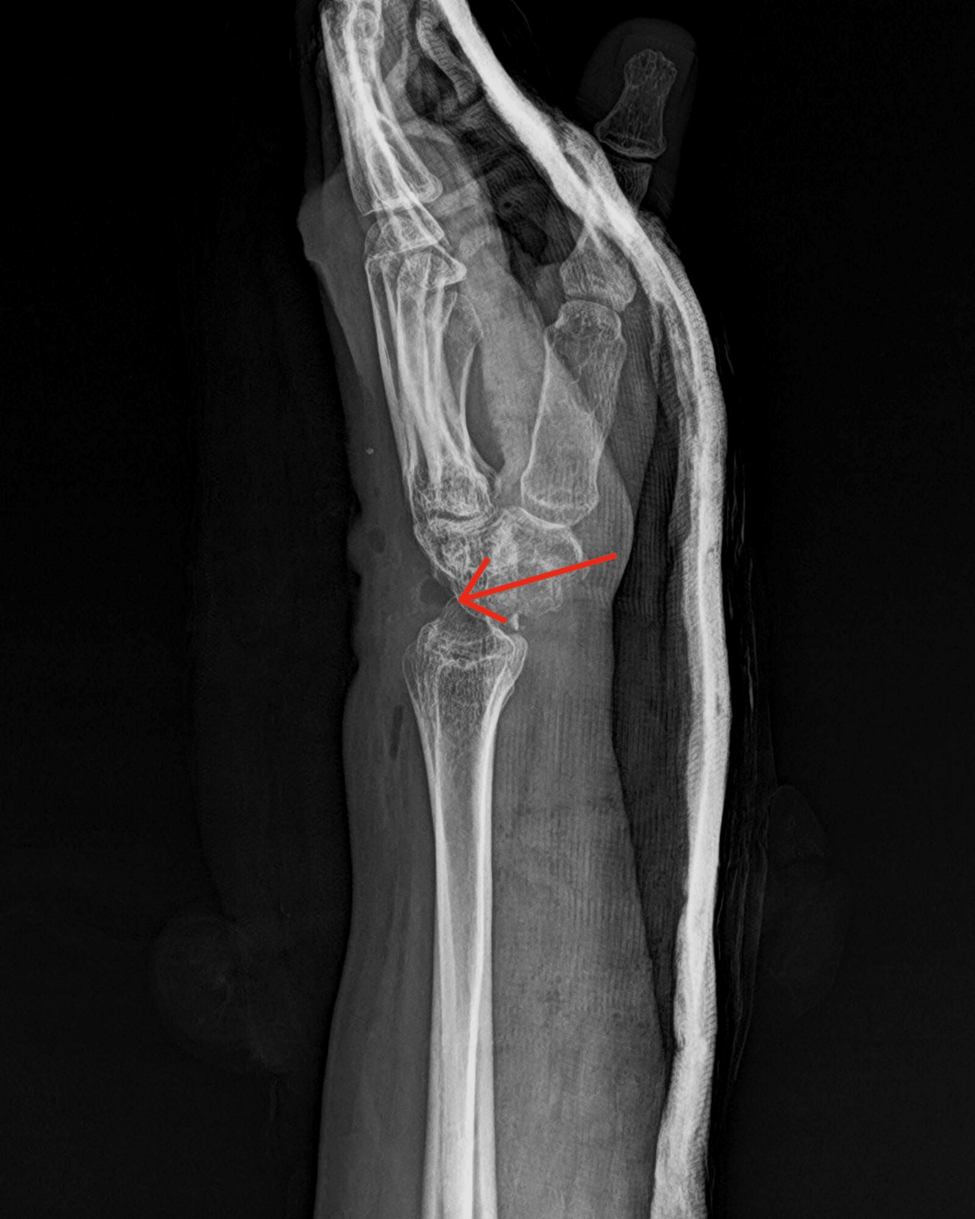
Post-operative radiological findings The plain X-ray in the lateral view shows the absence of the proximal carpal bones, likely due to a partial carpectomy.

**Figure 3:**
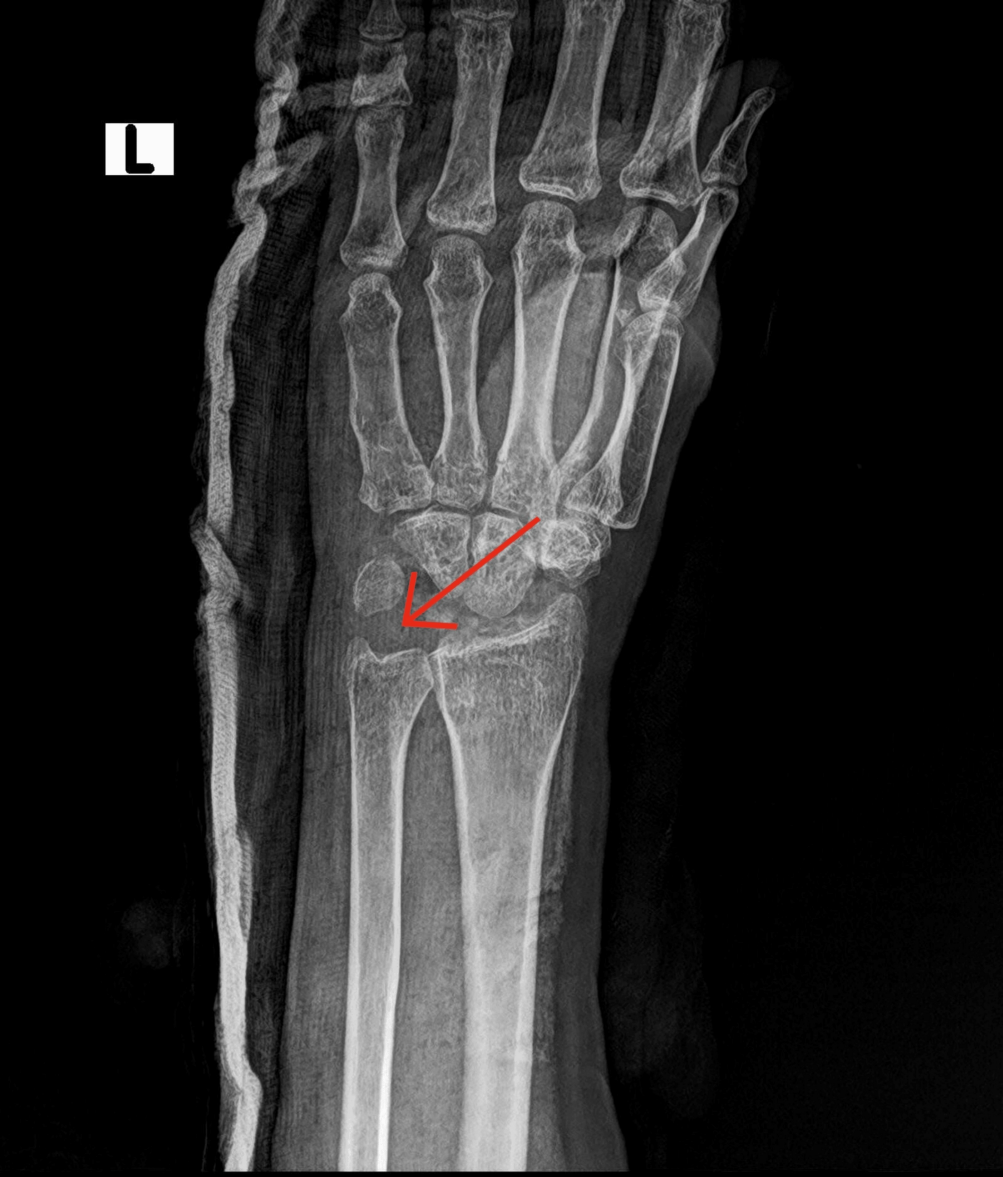
Post-operative radiological findings The plain X-ray in the AP view shows the absence of the proximal carpal bones, indicating a probable partial carpectomy.

The patient was kept NPO for two hours post-surgery to allow for recovery from anesthesia. Intravenous fluids were administered at a rate of 100 mL/hour using NS or RL to maintain hydration. Pain management included 1 g of Injection Paracetamol, which was given three times a day (TDS), and 75 mg of Injection Diclofenac, which was administered once daily in 100 mL of NS to manage inflammation and pain. To prevent infection, 1.5 g of Injection Cefuroxime was administered intravenously twice daily. Additionally, 40 mg of Injection Pantoprazole was given twice daily intravenously to prevent gastric acid-related complications. Oxygen supplementation was not required, indicating stable respiratory status. The post-operative radiological examination is shown in Figure 2 and Figure 3.

Post-operative treatment plan

On day 1, the patient received Capsule Pan-D (Pantoprazole 40 mg + Domperidone 30 mg) in the morning to prevent gastric acid-related issues and nausea and Signoflam (Aceclofenac + Paracetamol + Serratiopeptidase) tablet-form twice daily for pain relief, inflammation reduction, and to aid in healing. From days 2 to 4, the treatment included intravenous administration of 2 g of Injection Reflin (Cefazolin) twice daily as an antibiotic, 40 mg of Injection Pan (Pantoprazole) once daily for gastric protection, 4 mg of Injection Emeset (Ondansetron) twice daily to prevent nausea, and 100 mg of Injection Tramadol tablet-form twice daily for pain management. Additionally, Chymoral Forte (Trypsin-Chymotrypsin) tablet form was given twice daily from days 3 to 5 to reduce inflammation and promote healing. On days 4 and 5, 500 mg of Secrox (Cefuroxime) tablet form was administered twice daily as an antibiotic. On day 5, the patient also received 40 mg of Pan (Pantoprazole) tablet form in the morning, Vitamin C tablet form in the morning to boost immunity and aid healing, and Zincovit tablet form twice daily to support overall recovery, and all these are depicted in Table 7. This comprehensive treatment plan ensured effective pain management, infection prevention, gastric protection, and overall recovery.

**Table 7:** Drug Treatment *The asterisk in the table indicates that the drug is administered on the specified day. AU, arbitrary units; Cap - capsule; Inj - injection; IV - intravenous; P/O - per oral; ROA - routes of administration; T - tablet

Brand name	Generic name	Dose	ROA	Frequency	Duration with dates				
					Day 1	Day 2	Day 3	Day 4	Day 5
Cap. Pan-D	Pantoprazole + Domperidone	40 + 30 mg	P/O	1-0-0	*	-	-	-	-
T. Signoflam	Aceclofenac + Paracetamol + Serratiopeptidase	100 + 325 + 15 mg	P/O	1-0-1	*	-	-	*	*
Inj. Reflin	Cefazolin	2 g	IV	1-0-1	-	*	*	*	-
Inj. Pan	Pantoprazole	40 mg	IV	1-0-0	-	*	*	*	-
Inj. Emeset	Ondansetron	4 mg	IV	1-0-1	-	*	*	*	-
Inj. Tramadol	Tramadol	100 mg	IV	1-0-1	-	*	*	*	-
T. Chymoral Forte	Trypsin-Chymotrypsin	100,000 AU	P/O	1-0-1	-	-	*	*	*
T. Pan	Pantoprazole	40 mg	P/O	1-0-0	-	-	-	-	*
T. Secrox	Cefuroxime	500 mg	P/O	1-0-1	-	-	-	*	*
T. Vitamin C	Vitamin C	-	P/O	1-0-0	-	-	-	-	*
T. Zincovit	Zinc	-	P/O	1-0-0	-	-	-	-	*

Discharge summary

Throughout the hospital stay, the patient received regular dressings and physiotherapy, leading to notable symptomatic improvement. At discharge, the patient was advised to continue with Capsule Pan-D (Pantoprazole 40 mg + Domperidone 30 mg) once daily in the morning to manage gastric acid-related issues and nausea. Additionally, the patient was prescribed Pantoprazole 40 mg tablet form twice daily, Vitamin C tablet form once daily, Zincovit tablet form once daily, Shelcal tablet form 500 mg once daily for bone health, and Acton OR tablet form twice daily to address potential dehydration and electrolyte imbalances. These adjustments reflect the shift from intensive hospital treatment to a supportive home care regimen aimed at ensuring continued recovery and overall well-being.

## Discussion

Kienbock's disease presents a unique challenge in orthopedic and rheumatological practice due to its rarity and complex management requirements. This case highlights several critical aspects of Kienbock's disease, from diagnosis to treatment, contributing valuable insights to the broader understanding of this condition. The management of Kienbock's disease, particularly through PRC, has been the subject of extensive research. This case corroborates findings from El-Mowafi et al. [7], which noted significant improvements in pain relief and wrist function following PRC. PRC, involving the removal of the lunate and adjacent proximal row carpal bones, is often employed when conservative treatments fail to alleviate symptoms in the advanced stages of the disease. The improvements observed in this case support the continued use of PRC as a viable option for managing advanced Kienbock's disease. However, Chim et al. [8] emphasize the need for a comprehensive understanding of PRC's long-term outcomes and potential complications. Our case adds to this discourse by providing additional data on the procedure’s durability and effectiveness over time. Notably, while PRC has demonstrated efficacy in pain relief and functional improvement, its long-term benefits and risks necessitate further study to ensure optimal patient outcomes. Accurate diagnosis of Kienbock's disease is crucial for effective management. The use of advanced MRI techniques in this case, as highlighted by Allan et al. [9], proves essential for early and precise diagnosis. MRI offers superior sensitivity in detecting early bone marrow edema and subtle changes in the lunate, which X-rays often fail to reveal. This advanced imaging approach aligns with current best practices, underscoring the limitations of traditional radiographic methods and advocating for the integration of MRI in diagnostic protocols. The literature reflects a shift toward more sophisticated diagnostic methods. Earlier studies predominantly relied on X-rays, which often missed early-stage changes and led to delayed diagnoses. Our case supports the growing body of evidence favoring MRI and other advanced imaging techniques for improved diagnostic accuracy, thereby facilitating timely and appropriate interventions.

The staged treatment approach, guided by the Lichtman classification system, remains a cornerstone in managing Kienbock's disease. This case highlights the importance of tailoring interventions based on disease severity, as outlined by Chojnowski et al. [10]. For early stages (Lichtman stages I and II), conservative measures, such as wrist immobilization, anti-inflammatory medications, activity modification, and physical therapy, are employed to alleviate symptoms and prevent disease progression. These approaches aim to reduce inflammation and stabilize the wrist, thereby addressing early-stage issues effectively. As the disease progresses (stages III and IV), more invasive treatments become necessary. Core decompression, vascularized bone grafting, and radial shortening osteotomy are commonly considered for intermediate to advanced stages. This case’s progression to PRC aligns with established treatment protocols for advanced Kienbock’s disease. PRC addresses significant lunate collapse and carpal misalignment by removing the affected bones, thereby alleviating chronic pain and improving wrist function. The decision to perform PRC in this case underscores the importance of a staged approach and supports its application in similar cases. Managing Kienbock’s disease involves several challenges, including accurate diagnosis, effective treatment selection, and addressing patient-specific factors. This case illustrates the complexity of treatment decisions, particularly when transitioning from conservative to surgical interventions. The insights gained emphasize the need for a personalized approach, considering the disease’s progression and the patient’s response to initial treatments. Furthermore, this case highlights the importance of interdisciplinary collaboration in managing rare conditions. Orthopedic surgeons, radiologists, and rheumatologists must work together to ensure comprehensive care and optimize patient outcomes. The lessons learned from this case can guide clinicians in addressing similar challenges and refining treatment strategies for Kienbock’s disease.

This case report contributes to the broader understanding of Kienbock’s disease by providing a detailed account of the diagnostic and treatment process. It underscores the significance of advanced imaging techniques and a staged treatment approach in managing this rare condition. For clinicians, the case highlights the importance of accurate diagnosis and individualized treatment plans, particularly in advanced stages where surgical interventions may be required. Future research should focus on exploring innovative therapies and preventive measures for Kienbock’s disease. Long-term studies evaluating the efficacy and safety of PRC and other surgical options are essential to refine treatment strategies and improve patient outcomes. Additionally, further research into the pathophysiology of Kienbock’s disease could provide insights into preventive measures and potential new treatment approaches. This case report provides valuable insights into the management of Kienbock’s disease, particularly emphasizing the efficacy of PRC in advanced stages. It highlights the need for accurate diagnosis, a staged treatment approach, and interdisciplinary collaboration. By documenting the patient’s clinical journey, this report aids in refining treatment strategies and contributes to the broader understanding of Kienbock’s disease, ultimately enhancing clinical practices and stimulating further research.

## Conclusions

In conclusion, this case of Kienbock's disease highlights the importance of accurate diagnosis and tailored treatment. The use of advanced imaging and the Lichtman classification system facilitated an effective management plan, culminating in a successful PRC. This case underscores the necessity for early detection and individualized care in improving patient outcomes and contributes valuable insights into managing this rare condition.
